# Identification of Common Pathogenetic Processes between Schizophrenia and Diabetes Mellitus by Systems Biology Analysis

**DOI:** 10.3390/genes12020237

**Published:** 2021-02-07

**Authors:** Md Rezanur Rahman, Tania Islam, Ferdinando Nicoletti, Maria Cristina Petralia, Rosella Ciurleo, Francesco Fisicaro, Manuela Pennisi, Alessia Bramanti, Talip Yasir Demirtas, Esra Gov, Md Rafiqul Islam, Bashair M. Mussa, Mohammad Ali Moni, Paolo Fagone

**Affiliations:** 1 Department of Biotechnology and Genetic Engineering, Faculty of Biological Sciences, Islamic University, Kushtia 7003, Bangladesh; rezanur12@yahoo.com; 2 Department of Biochemistry and Biotechnology, Khwaja Yunus Ali University, Enayetpur, Sirajganj 6751, Bangladesh; taniaislam1304@gmail.com; 3 Department of Biomedical and Biotechnological Sciences, University of Catania, 95124 Catania, Italy; drfrancescofisicaro@gmail.com (F.F.); Manuela.pennisi@unict.it (M.P.); paolofagone@yahoo.it (P.F.); 4 IRCCS Centro Neurolesi “Bonino-Pulejo”, Via Provinciale Palermo, Contrada Casazza, 98124 Messina, Italy; m.cristinapetralia@gmail.com (M.C.P.); rossella.ciurleo@irccsme.it (R.C.); alessia.bramanti@irccsme.it (A.B.); 5 Department of Bioengineering, Faculty of Engineering, Adana Alparslan Turkes Science and Technology University, Adana 01250, Turkey; tyasird@gmail.com (T.Y.D.); egov@atu.edu.tr (E.G.); 6 School of Biomedical Sciences, Faculty of Health, Institute of Health and Biomedical Innovation, Queensland University of Technology (QUT), Brisbane, QLD 4059, Australia; rafiqulislambd7@gmail.com; 7 Department of Pharmacy, Faculty of Biological Science and Technology, Jashore University of Science and Technology, Jashore 7408, Bangladesh; 8 Basic Medical Sciences Department, College of Medicine, University of Sharjah, Sharjah P.O. Box 27272, United Arab Emirates; bmussa@sharjah.ac.ae; 9 WHO Collaborating Centre on eHealth, UNSW Digital Health, School of Public Health and Community Medicine, Faculty of Medicine, Sydney, NSW 2052, Australia; m.moni@unsw.edu.au

**Keywords:** schizophrenia, type 2 diabetes mellitus, differentially expressed genes, pathways, transcription factors

## Abstract

Schizophrenia (SCZ) is a psychiatric disorder characterized by both positive symptoms (i.e., psychosis) and negative symptoms (such as apathy, anhedonia, and poverty of speech). Epidemiological data show a high likelihood of early onset of type 2 diabetes mellitus (T2DM) in SCZ patients. However, the molecular processes that could explain the epidemiological association between SCZ and T2DM have not yet been characterized. Therefore, in the present study, we aimed to identify underlying common molecular pathogenetic processes and pathways between SCZ and T2DM. To this aim, we analyzed peripheral blood mononuclear cell (PBMC) transcriptomic data from SCZ and T2DM patients, and we detected 28 differentially expressed genes (DEGs) commonly modulated between SCZ and T2DM. Inflammatory-associated processes and membrane trafficking pathways as common biological processes were found to be in common between SCZ and T2DM. Analysis of the putative transcription factors involved in the regulation of the DEGs revealed that STAT1 (Signal Transducer and Activator of Transcription 1), RELA (v-rel reticuloendotheliosis viral oncogene homolog A (avian)), NFKB1 (Nuclear Factor Kappa B Subunit 1), and ERG (ETS-related gene) are involved in the expression of common DEGs in SCZ and T2DM. In conclusion, we provide core molecular signatures and pathways that are shared between SCZ and T2DM, which may contribute to the epidemiological association between them.

## 1. Introduction

Schizophrenia (SCZ) is a psychiatric disorder characterized by psychotic events in a continuous and/or relapsing mode. Compared to the general population, SCZ patients are reported with a 1.5–2 times higher risk of type 2 diabetes (T2DM) [1]. Several factors, including the environment, the use of antipsychotic medications, and genetic predisposition, may explain this epidemiological association [1,2,3,4]. People with serious psychiatric disorders live sedentary lives and smoke more often than the general population, which are considered as risk factors of T2DM [4]. Antipsychotic medicines are also found to trigger metabolic adversity that leads to a dramatic increase in body weight [5]. Multiple reports have demonstrated a link between antipsychotic medications and the likelihood of developing T2DM [6,7,8], but this still needs further confirmation [4]. Multiple etiopathogenetic mechanisms seem to be involved in the association between SCZ and T2DM. The genetic contribution of SCZ in the early onset of T2DM has been investigated and several genes were identified by genome-wide association studies [9,10,11,12]. Although genetic predispositions are recognized, it is believed that environmental, neurological, and metabolic processes may contribute to the increased risk of developing T2DM by SCZ patients. However, the pathogenetic mechanisms of nongenetic variants of SCZ and T2DM still need to be explored.

Alterations of the transcriptome have lately been explored to characterize the molecular and cellular processes in complex diseases [13,14]. Significant numbers of studies have independently characterized the gene expression signatures of SCZ [15,16,17] and T2DM [18,19], but no attempt has been made to establish shared gene signatures, associated regulators, and biological processes between SCZ and T2DM. Therefore, the molecular signatures and pathways associated with an increased T2DM risk in SCZ remain unclear. In this study, we integrated peripheral blood mononuclear cell (PBMC) transcriptomic meta-analysis data and systems biology to investigate SCZ and T2DM molecular interactions and pathways that may offer new insights into the shared pathogenetic mechanisms of SCZ and T2DM. The phenotypic and functional analysis of PBMCs has widely been used as a tool to study the etiopathogenetic mechanisms underlying several disorders, including SCZ and diabetes [20,21]. In particular, the transcriptomic analyses of these cells may allow the identification of commonly altered DEG in these two diseases, thus allowing the initial identification of cellular and molecular pathways that are abnormally expressed in SCZ and diabetes. This could allow the identification of specific cellular or soluble biomarkers that may be useful to predict therapeutic responses and help to design tailored therapeutic approaches.

**Figure 1:**
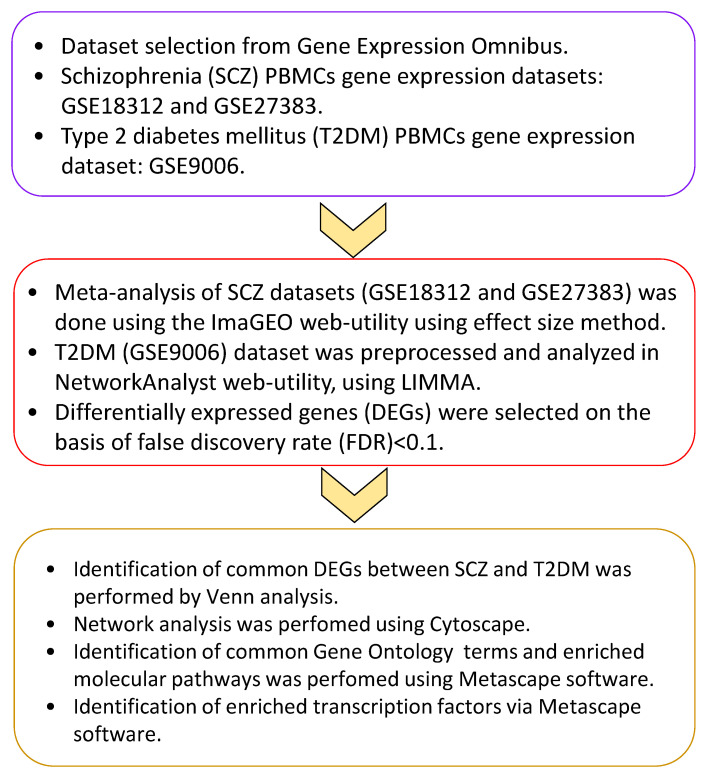
The workflow used in this study.

In order to shed light on the possible shared pathways of SCZ and T2DM pathogenesis, functional annotation and transcription factor (TF) analysis was conducted in the present study (Figure 1).

## 2. Materials and Methods

### 2.1. Acquisition of Blood Transcriptomic Data

In order to obtain suitable datasets of SCZ and T2DM, we queried the transcriptomics database Gene Expression Omnibus (GEO). We searched the database using the following keywords: “schizophrenia”, “blood”, and “*Homo sapiens*”. Inclusion criteria for the selection of the datasets were as follows: (i) whole-genome gene expression data; (ii) the datasets should contain both cases and matched controls; (iii) human peripheral blood mononuclear cell samples. For SCZ, we found the two datasets, GSE18312 [22,23] and GSE27383 [24]. GSE18312 contained peripheral blood mononuclear cell (PBMC) gene expression (messenger RNAs (mRNAs)) of 13 SCZ cases and eight healthy controls. GSE27383 contained gene expression profiling of PBMC samples from 43 SCZ cases and 29 controls. Similarly, we queried the GEO database with the above search criteria for T2DM datasets, and the only available dataset was GSE9006 [20], which contained gene expression profiling of 12 T2DM cases and 24 controls from PBMCs samples. The characteristics of the datasets are presented in Table 1.

### 2.2. Analysis of Transcriptomic Data

We performed a meta-analysis of the two SCZ PBMC datasets (GSE18312 and GSE27383) using the effect size method via ImaGEO web-utility [25] as described elsewhere [26]. From the meta-analysis, we selected the differentially expressed genes (DEGs) in SCZ PBMCs compared to controls. GSE9006 was analyzed to identify DEGs using the LIMMA method in R [27] as implemented in NetworkAnalyst [28]. For the normalization of the dataset, we employed the variance stabilizing normalization (VSN) algorithm [29], followed by quantile normalization [30]. The significant genes were selected on the basis of a false discovery rate (FDR) < 0.1. The adjustment of the *p*-value was done by the Benjamini–Hochberg method. The total number of shared genes among the datasets was 11,112, which were considered for all the analyses.

To evaluate the significance of the overlap between the DEGs belonging to the SCZ and T2DM signatures, a one-tailed chi-square test was performed, using the total number of shared genes among the datasets as the background [31]. The representation factor is defined as the number of overlapping genes divided by the expected number of overlapping genes drawn from the two groups. A representation factor >1 indicates more overlap than expected, while a representation factor <1 indicates less overlap than expected. A *p*-value < 0.05 was considered to be statistically significant.

### 2.3. Functional Insights into the Significant Genes

For the gene ontology and enrichment analysis, we utilized the bioinformatics tool “Metascape” [32]. By default, the enrichment analysis performed by Metascape makes use of data sources, including gene ontology, KEGG (Kyoto Encyclopedia of Genes and Genomes), and Reactome. Metascape clusters enriched terms into nonredundant groups. In each cluster, Metascape selects the most important (lowest *p*-value) term to represent the cluster in the bar graph. The hypergeometric test was performed to screen significant terms. A Bonferroni corrected *p*-value < 0.05 was considered for significant term selection.

### 2.4. Network Analysis

The GeneMania database [33] was used to construct a network of the common DEGs between SCZ and T2DM. Interaction data included physical interaction, co-expression, prediction, co-localization, pathway, genetic interactions, and shared protein domains. GeneMania assigns weights in order to maximize the connectivity between all input genes. A maximum of 20 resultant genes and a maximum of 10 attributes are considered, by default [33]. The Cytoscape software [34] was used for visualization of the network and to perform network analysis, using the NetworkAnalyzer utility. Topological analysis was performed considering the network as undirected (i.e., containing only undirected edges). Hubs were defined as the top 20% of nodes with the highest degree of centrality, which corresponds to the number of edges linked to each given node.

## 3. Results

### 3.1. Identification of Common Transcriptional Signatures between SCZ and T2DM PBMCs

First of all, we performed a transcriptomic meta-analysis of two SCZ PBMCs datasets (with accession numbers GSE18312 and GSE27383) obtained from the GEO database. The meta-analysis identified 354 significant DEGs at FDR < 0.1. The complete list of DEGs is presented in Table S1 (Supplementary Materials). Secondly, we analyzed the PBMC transcriptomic dataset of T2DM (with accession number GSE9006). The analysis revealed 678 significant DEGs between T2DM and healthy controls (FDR < 0.1). The complete list of DEGs characterizing T2DM PBMCs is presented in Table S2 (Supplementary Materials).

**Figure 2:**
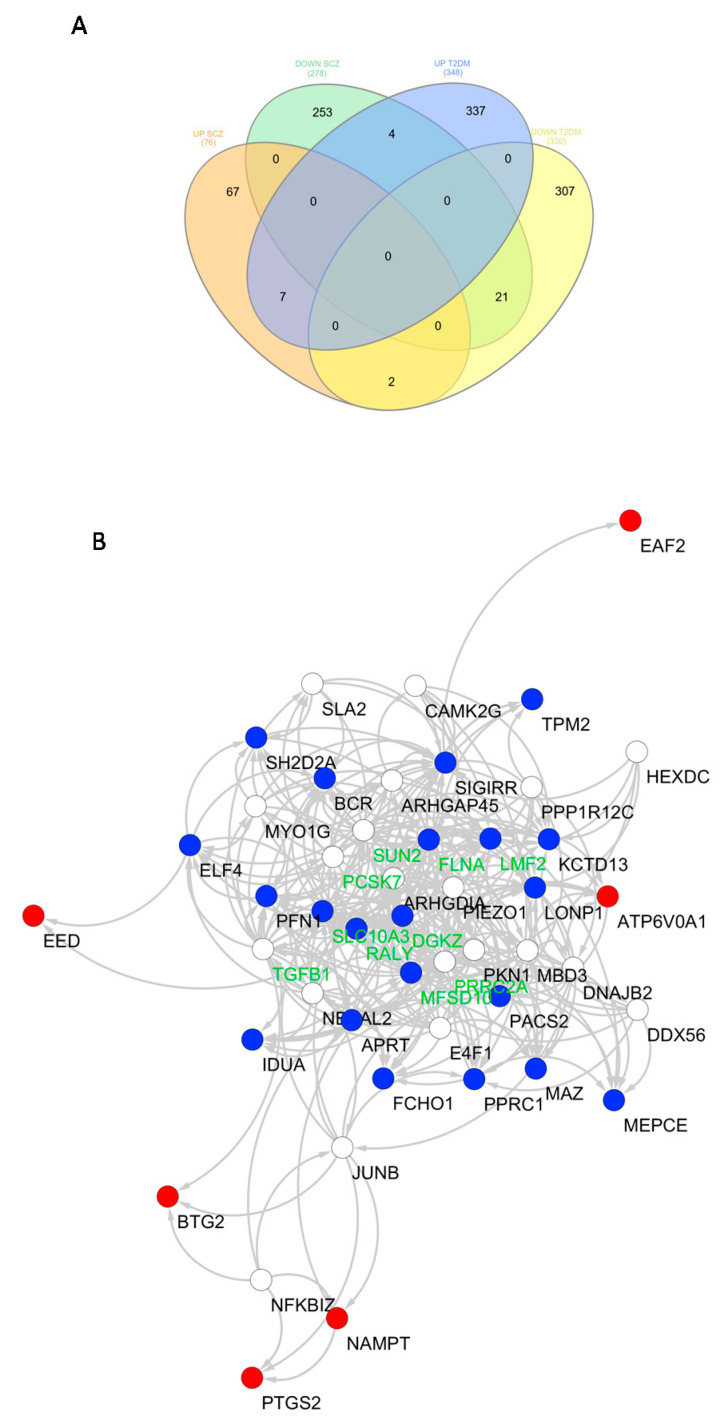
The common differentially expressed genes and pathways between schizophrenia (SCZ) and type 2 diabetes mellitus (T2DM). (**A**) Venn diagram representing common differentially expressed genes (DEGs) between SCZ and T2DM, where up denotes upregulated and down denotes downregulated DEGs. (**B**) A functional network was constructed using the 28 common DEGs between SCZ and T2DM, using the GeneMania prediction server. Overall, the network included 47 nodes. Nodes colored in red represent the commonly upregulated DEGs, while nodes in blue represent the commonly downregulated DEGs between SCZ and T2DM. Nodes labeled in green represent the hub genes of the network.

Our analysis showed a significant number of overlapping DEGs between SCZ and T2DM PBMCs (Table 2). In particular, we found seven common upregulated DEGs (*p* = 0.01 using a one-tailed chi-square test; representation factor = 2.9) (*BTG2*, *EED*, *HBP1*, *PTGS2*, *NAMPT*, *ATP6V0A1*, and *EAF2*) between SCZ and T2DM. Our analysis also showed that 21 downregulated DEGs (*LONP1*, *RALY*, *PACS2*, *SH2D2A*, *DGKZ*, *MEPCE*, *KCTD13*, *ELF4*, *MFSD10*, *MAZ*, *SIGIRR*, *FCHO1*, *BCR*, *PPRC1*, *TPM2*, *IDUA*, *PFN1*, *LMF2*, *FLNA*, *APRT*, and *SLC10A3*) were common between SCZ and T2DM (*p* < 0.0001 using a one-tailed chi-square test; representation factor = 2.5) (Figure 2). On the other hand, two genes were found to be upregulated in SCZ and downregulated in T2DM (*p* = 0.394 using a one-tailed chi-square test; representation factor = 0.9) (*TECR* and *HNRNPK*), while four genes were found to be downregulated in SCZ and upregulated in T2DM (*p* = 0.061 using a one-tailed chi-square test; representation factor = 0.5) (*RIC8B*, *CSRNP2*, *ASTE1*, and *SLA*).

Network analysis was performed on the 28 commonly regulated DEGs between SCZ and T2DM. According to the functional similarity and shared properties, 19 genes were predicted to interact with the initial 28 DEGs and are presented in the functional association network in Figure 2B. Network analysis identified 10 hub genes, i.e., *FLNA*, *PRRC2A*, *MFSD10*, *RALY*, *TGFB1*, *LMF2*, *PCSK7*, *SUN2*, *DGKZ*, and *ARHGDIA.*

### 3.2. Identification of Common Functional Gene Ontology Terms in SCZ and T2DM PBMCs

**Figure 3:**
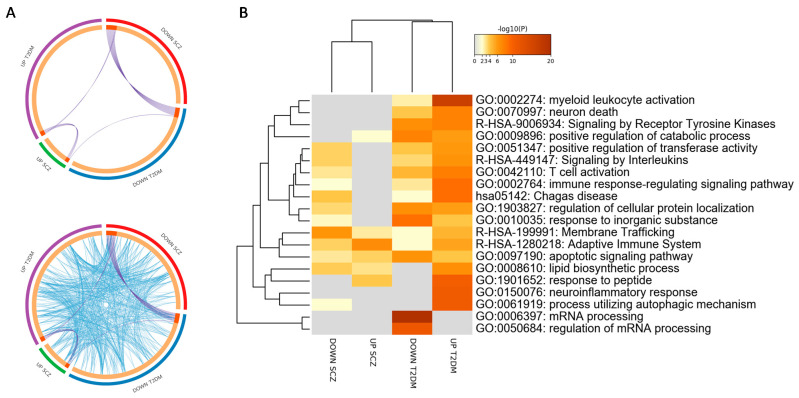
Functional annotations performed on the differentially expressed genes in schizophrenia (SCZ) and type 2 diabetes mellitus (T2DM) peripheral blood mononuclear cells (PBMCs). (**A**) Circos plot showing overlapping between the differentially expressed genes (DEGs) in SCZ and T2DM PBMCs. Purple lines link the same genes that are shared by the input lists. Blue lines link the different genes that fall in the same ontology term. (**B**) Hierarchical clustering of the top 20 most enriched terms among the DEGs in SCZ and T2DM PBMCs. The heatmap is colored by the *p*-values, and gray cells indicate the lack of significant enrichment.

To shed light on the biological insights of DEGs, we carried out functional enrichment analysis to identify gene ontologies and molecular pathways enriched by the common DEGs. Several gene ontology terms were found in common between SCZ and T2DM (Figure 3). Among the most significant terms enriched by the upregulated DEGs, which were in common between SCZ and T2DM, we found “positive regulation of catabolic process”, “regulation of binding”, “membrane trafficking”, “adaptive immune systems”, and “apoptotic signaling pathway” (Figure 3B).

On the other hand, among gene terms enriched by the downregulated DEGs in SCZ and T2DM, we found “lymphocyte activation”, “signaling by interleukins”, “regulation of cellular protein localization”, “positive regulation of transferase activity”, “asparagine *N*-linked glycosylation”, “membrane trafficking”, “adaptive immune systems”, and “apoptotic signaling pathway” (Figure 3B). It is interesting to note that “membrane trafficking”, “adaptive immune systems”, and “apoptotic signaling pathway” were common in both upregulated and downregulated DEGs between SCZ and T2DM (Figure 3B and Figure S1, Supplementary Materials).

### 3.3. Prediction of Transcription Factor Overlapping between SCZ and T2DM PBMCs

**Figure 4:**
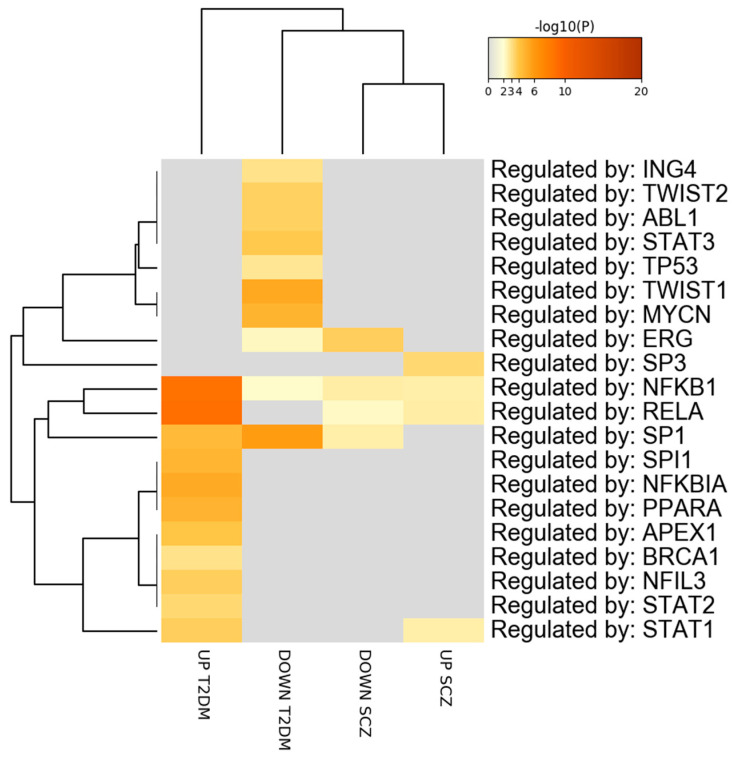
Putative transcription factors regulating the differentially expressed genes in peripheral blood mononuclear cells (PBMCs) from schizophrenia (SCZ) and type 2 diabetes mellitus (T2DM). The transcription factors are visualized as a hierarchical clustering. The heatmap is colored by the *p*-values, and gray cells indicate the lack of significant enrichment.

Analysis of the putative transcription factors involved in the regulation of the DEGs modulated in SCZ and T2DM revealed that STAT1, RELA, and NFKB1 are involved in the expression of common upregulated genes in SCZ, and T2DM. ERG was detected as regulator of the common downregulated genes in SCZ and T2DM (Figure 4; Table S3, Supplementary Materials).

## 4. Discussion

Despite the effort of genome-wide association studies to detect the genetic contribution of SCZ in T2DM, the molecular mechanisms of T2DM comorbidity in a subset of SCZ patients remains to be deciphered [9,10,11,12]. Hackinger et al. identified 29 genes that were associated with both T2DM and SCZ, using a genome-wide association approach [9]. Purcell and coworkers performed SCZ risk analysis, but did not find any significant correlation [10]. Another study also investigated the genetic risk of SCZ and detected a weak link between risk of SCZ score and T2DM [11]. It should be noted that none of the previously described genes were found to be modulated in our analysis. The use of whole-genome transcriptomic analyses has largely been used in the past few years to study autoimmune disorders, cancer, and neurodegenerative and neuropsychiatric diseases [17,35,36,37,38], in order to shed light on their pathogenetic mechanisms [39,40,41] and to identify potential therapeutic targets [42,43,44,45]. In the present study, we showed a common transcriptomic signature between SCZ and T2DM, suggesting potential overlapping pathogenetic processes. A number of genes that were found to overlap between SCZ and T2DM have already been associated with either one of these two disorders, particularly *DGKZ, APRT, KCTD13*, and *PTGS2* for *SCZ* and *RALY, FLNA, NAMPT, PTGS2, BCR, APRT*, and *DGKZ* for T2DM, as reported in the DisGeNET database (https://www.disgenet.org/ on 10 January 2021). Interestingly, DGKZ, APRT, and PTGS2 are commonly associated with both diseases. The common upregulated DEGs were enriched in the “positive regulation of catabolic process” pathway, which is implicated in the T2DM and low-grade inflammation as manifested by the insulin resistance mechanism observed in T2DM patients [46]. Insulin resistance inhibits the uptake of glucose by adipocytes and muscle cells and prevents glucose synthesis in hepatic cells, suggesting a prominent feature of catabolic processes in T2DM. Our analysis also showed the enrichment of “membrane trafficking” as a shared process in both SCZ and T2DM. Membrane trafficking is a process ubiquitously found in all types of tissues, and perturbation of membrane trafficking is involved in various disorders including T2DM, neuropsychiatric, immunological, systemic, and multisystem disorders [47]. Emerging data indicate that clathrin-mediated endocytosis, a key mechanism of the cellular membrane and protein-trafficking systems, may be involved in psychosis, SCZ, and bipolar disorder [48]. Previous studies have also shown that several crucial genes of SCZ are involved in cellular processes linked to cellular and membrane trafficking systems, and that these trafficking systems affect synaptic dysfunction [48]. Indeed, compelling evidence has suggested synaptic dysfunction as causative for several neuropsychiatric disorders [49]. It is hypothesized that dysregulated synaptic development and plasticity are involved in the pathogenesis of SCZ and autism spectrum disorder [49]. Many antipsychotic drugs can affect the proteins of clathrin-mediated trafficking processes, suggesting the possibility to design drugs that may influence membrane trafficking in SCZ and T2DM.

In agreement with previous reports, our study identified that pathways related to the immune system are involved in both SCZ and T2DM [50,51]. Our study identified the “signaling by interleukins” pathway to be enriched, which is notably involved in insulin resistance in T2DM and in SCZ [52]. The impact of the immune system in SCZ pathology is suggested by the epidemiological observation of the increased risk of SCZ patients to develop immune-mediated disorders. Genetic associations have been reported between SCZ and Crohn’s disease, ulcerative colitis, multiple sclerosis, psoriasis, and systemic lupus erythematosus [53]. More specifically, Pouget et al. identified 581 variants (563 non-HLA variants and 18 HLA variants) that were associated with immune-mediated disorders at genome-wide significance [53]. In T2DM, a large body of data has also pointed out the role for cytokines in promoting local and systemic inflammation, which may, therefore, represent critical players in the development and maintenance of insulin resistance [54]. In particular, the IL-1 (Interleukin-1) family of cytokines has been linked to obesity-induced adipose tissue inflammation and T2DM. High plasma IL-1β levels have been associated with an increased risk of developing T2DM [55], and mice lacking the inflammasome, IL-1β, and receptor IL-1R1 are protected from the development of T2DM [56]. Accordingly, a multicenter, open-label, randomized controlled trial investigating the effects of the IL-1 inhibitor Anakinra in T2DM patients showed a significant reduction in the HbA1c (glycated hemoglobin A1c) %, after correcting for clinical confounders, such as sex, age, disease duration, use of oral antidiabetic drug, and body mass index [57]. Moreover, an involvement of the apoptotic signaling pathway was identified in the present study. Interestingly, an activated complement system and caspase-independent apoptosis were found in leukocytes from SCZ patients, supporting a link between SCZ and immune dysregulation and suggesting the presence of apoptotic processes in leukocytes [58]. Along the same lines, expressions of proapoptotic markers (i.e., Caspase-3, Fas, and Bax-BCL2 Associated X) were significantly higher, while reduced expression of the antiapoptotic marker Bcl-2 (B-cell lymphoma 2) was observed in lymphocytes of T2DM patients [59].

Next, we analyzed the putative TFs that may regulate the expression (i.e., transcription) of common DEGs in PBMCs from SCZ and T2DM patients. Among the identified TFs, RELA and NFKB1 were enriched for the upregulated DEGs of both SCZ and T2DM. It is generally assumed that aberrant immune and inflammatory responses are involved in SCZ and T2DM. Nuclear factor kappa B (NF-κB) has a very crucial role in immune and inflammatory processes, and RELA encodes a major component of the NF-κB complex. A previous report showed that three SNPs (rs11820062, rs2306365, and rs7119750) in RELA gene are associated with SCZ [60]. NFKB1 is a component of the NF-κB family. Previous findings also reported a close association between cytokine expression and NF-κB activation in SCZ [61], which suggests that changes in cytokines expression and the NF-κB mediated cascade might contribute to the pathogenesis of SCZ. Furthermore, the NF-κB pathway plays significant role in the pathogenesis of T2DM and its associated complications [62,63]. Additionally, STAT1 has been predicted by TRRUST (Transcriptional Regulatory Relationships Unraveled by Sentence-based Text mining) analysis. The involvement of STAT1 is supported by data showing the activation of the IL23/JAK/STAT (Interleukin-23/ Janus Kinase/Signal Transducer and Activator of Transcription) pathway in T2DM PBMCs [64] and the activation of STAT1 in SCZ, which negatively correlated with cognitive performance [65].

On the other hand, the ERG transcription factor [66] was identified as implicated in the regulation of the downregulated DEGs in both SCZ and T2DM. ERG has been found to be required for hematopoiesis, hematopoietic stem-cell function, and the maintenance of normal platelet numbers [67], and it acts as an oncogene in leukemias, as well as solid tumors, such as prostate cancer [68]. This is the first report associating ERG with either SCZ or T2DM, and further studies need to be carried out for the evaluation of the role of this transcription factor in the etiopathogenesis of these disorders.

Interestingly, our network analysis identified TGF (Transforming Growth Factor)-β to be implicated in both SCZ and T2DM. This is worth noting as SCZ and T2DM have been associated with activated peripheral and central inflammatory responses [69,70]. As previously reported, despite the presence of increased serum levels of TGF-β in SCZ [71] and T2DM patients [72], no significant modulation or, more strikingly, a downregulation in the expression of this cytokine can be observed in PBMCs [73,74]. Along the same lines, in our study, the expression levels of TGF-β were not found to be significantly modulated in either of the two disorders (with only a trend of reduction—FDR = 0.068 for T2DM and FDR = 0.346 for SCZ). However, TGF-β resulted a central hub in the functional network constructed using the common DEGs between SCZ and T2DM. In particular, TGF-β was found to functionally interact with 17 out of the 47 genes belonging to the network (i.e., *PACS2, APRT, BCR, BTG2, DGKZ, ELF4, FCHO1, FLNA, IDUA, JUNB, MFSD10, MYO1G, PCSK7, PFN1, RALY, SLA2*, and *SLC10A3*). Notably, among the TGF-β-interacting genes, BTG2 (B-cell translocation gene 2), which interacts with the bone morphogenetic protein (BMP)-activated SMADs (Small Mother Against Decapentaplegic) [75], known to antagonize the effect of TGF-β, resulted commonly upregulated in our analysis. We may speculate that alteration in the SMAD-dependent signaling pathways could be partly responsible for either the onset or the progression of SCZ and T2DM and, hence, warrants further exploration. We are currently not able to determine whether the common transcriptomic signature that we herein characterized represents the consequence of the diseases or rather an ab initio genetic susceptibility factor that may promote the comorbidity of T2DM in SCZ patients. Functional studies on the role of the identified biological processes will be needed to dismantle their etiopathogenetic role and to exploit them for better pharmacological management of SCZ patients.

There are several limitations in the present study that need to be mentioned. The analysis was performed on PBMCs, which do not represent the main tissue of action for T2DM or SCZ. Therefore, further work should be done in order to identify common transcriptional changes affecting the pancreas and the central nervous system of both SCZ and T2DM patients. Moreover, our study involved a limited number of samples; hence, no adjustment for sex, age, and disease duration could be performed. Moreover, we have no data about whether a set of patients included in the analysis suffered from both diseases at the same time. Lastly, the datasets only shared 11,112 genes, and we could not determine whether the remaining genes were modulated in the two disorders.

## 5. Conclusions

This study aimed to provide novel molecular signatures and pathways that may underlie both SCZ and T2DM pathogenesis. Using a comprehensive systems biology analysis, we determined the molecular signatures and pathways via the reconstruction of comprehensive SCZ and T2DM specific biological networks. We revealed 28 genes concordantly dysregulated in SCZ and T2DM that may clarify genes that potentially promote the progression of T2DM in SCZ patients. Our study predicted STAT1, RELA, NFKB1, and ERG has regulators of the common DEGs between SCZ and T2DM. Immune systems, inflammatory-associated processes, and membrane trafficking pathways were prioritized as common biological processes in SCZ and T2DM. The new common genes and associated regulators, as well as biological processes, identified in this study can be a crucial resource for understanding the association between SCZ and T2DM and may help to develop a precision medicine approach.

## Figures and Tables

**Table 1:** Characteristics of the datasets used in this study.

Accession	Source/Tissue	Sample	Patients Characteristics	Healthy Controls Characteristics	Platform
Schizophrenia					
GSE18312	PBMCs	13 SCZ patients and 8 healthy controls	Age (years): 43.6 ± 8.6	Age (years): 44.6 ± 6.5	Affymetrix Human Exon 1.0 ST Array
			% female: 30.7	% female: 37.5	
			Race: European-American 38.4% Hispanic 15.3% African-American 46.2%	Race: European-American 65.5% Hispanic 12.5% Asian 12.5% African-American 12.5%	
GSE27383	PBMCs	43 SCZ subjects and 29 controls	Age (years): 23 ± 4	Age (years): 23.9 ± 4.1	Affymetrix Human Genome U133 Plus 2.0 Array
			Race: European 48.8% Surinamese/African 14.6% Cape Verdean 2.4% Surinamese/Hindustani 14.6% Moroccan/North African 4.9% Asian 2.4% Mixed 7.3% Unknown 4.9%	Race: European 82.6% Surinamese/African 3.4% Surinamese/Hindustani 3.4% Asian 3.4% Mixed 6.9%	
Type 2 diabetes mellitus					
GSE9006	PBMCs	12 T2DM patients and 24 healthy controls	Age (years): 14 ± 2.3	Age (years): 11.3 ± 4.6	Affymetrix Human Genome U133A Array
			% female: 58	% female: 58	
			Race: Caucasian 16.6% Hispanic 16.6% African-American 58.3% Asian 8.3%	Race: Caucasian 45.8% Hispanic 29.1% Mixed or unknown 25%	

PBMC: peripheral blood mononuclear cell; SCZ: Schizophrenia; T2DM: type 2 diabetes mellitus.

**Table 2:** Differentially expressed genes concordantly regulated in SCZ and T2DM PBMCs.

Genes Symbol	Description	Regulation
*BTG2*	BTG anti-proliferation factor 2	Upregulated
*EED*	embryonic ectoderm development	Upregulated
*HBP1*	HMG-box transcription factor 1	Upregulated
*PTGS2*	prostaglandin-endoperoxide synthase 2	Upregulated
*NAMPT*	nicotinamide phosphoribosyltransferase	Upregulated
*ATP6V0A1*	ATPase H+ transporting V0 subunit a1	Upregulated
*EAF2*	ELL associated factor 2	Upregulated
*LONP1*	lon peptidase 1, mitochondrial	Downregulated
*RALY*	RALY heterogeneous nuclear ribonucleoprotein	Downregulated
*PACS2*	phosphofurin acidic cluster sorting protein 2	Downregulated
*SH2D2A*	SH2 domain containing 2A	Downregulated
*DGKZ*	diacylglycerol kinase zeta	Downregulated
*MEPCE*	methylphosphate capping enzyme	Downregulated
*KCTD13*	potassium channel tetramerization domain containing 13	Downregulated
*ELF4*	E74 like ETS transcription factor 4	Downregulated
*MFSD10*	major facilitator superfamily domain containing 10	Downregulated
*MAZ*	MYC associated zinc finger protein	Downregulated
*SIGIRR*	single Ig and TIR domain containing	Downregulated
*FCHO1*	FCH domain only 1	Downregulated
*BCR*	BCR, RhoGEF and GTPase activating protein	Downregulated
*PPRC1*	peroxisome proliferator-activated receptor γ, coactivator-related 1	Downregulated
*TPM2*	tropomyosin 2	Downregulated
*IDUA*	iduronidase, α-L-	Downregulated
*PFN1*	profilin 1	Downregulated
*LMF2*	lipase maturation factor 2	Downregulated
*FLNA*	filamin A	Downregulated
*APRT*	adenine phosphoribosyltransferase	Downregulated
*SLC10A3*	solute carrier family 10 member 3	Downregulated

## Data Availability

All data are available in the Gene Expression Omnibus (GEO) database (https://www.ncbi.nlm.nih.gov/gds).

## Supplementary Materials

The following are available online at https://www.mdpi.com/2073-4425/12/2/237/s1: Figure S1. Network of enriched Gene Ontology terms; Table S1. List of the differentially expressed genes (DEGs) identified in SCZ PBMCs by meta-analysis of the GSE18312 and GSE27383 datasets; Table S2. List of the differentially expressed genes (DEGs) identified in T2DM PBMCs by analysis of the GSE9006 dataset; Table S3. Predicted transcription factors regulating the differentially expressed genes (DEGs) in SCZ and T2DM PBMCs, using the TRRUST database.
