# Supplementary material for: Identification of Common Pathogenetic Processes between Schizophrenia and Diabetes Mellitus by Systems Biology Analysis
Source: Genes (Basel). 2021 Feb 7;12(2):237. doi: 10.3390/genes12020237 (PMC7916024; doi:10.3390/genes12020237)
Supplement: Supplementary file 1 [file genes-12-00237-s001.zip › supplementary/Figure S1 Legend.docx]

**Suppl.Figure Legend**

**Suppl. Figure 1.** **Network of enriched Gene Ontology terms.** A. A subset of representative terms from the full cluster has been converted into a network layout. Each term is represented by a circle node, where its size is proportional to the number of input genes fall into that term, and its color represent its cluster identity. Terms with a similarity score > 0.3 are linked by an edge (the thickness of the edge represents the similarity score). The network is visualized with Cytoscape (v3.1.2) with “force-directed” layout and with edge bundled for clarity. One term from each cluster is selected to have its term description shown as label. B. The same enrichment network with its nodes displayed as pies. Each pie sector is proportional to the number of hits originated from a gene list.
