# Supplementary figures and images for: Identification of Common Pathogenetic Processes between Schizophrenia and Diabetes Mellitus by Systems Biology Analysis
Source: Genes (Basel). 2021 Feb 7;12(2):237. doi: 10.3390/genes12020237 (PMC7916024; doi:10.3390/genes12020237)

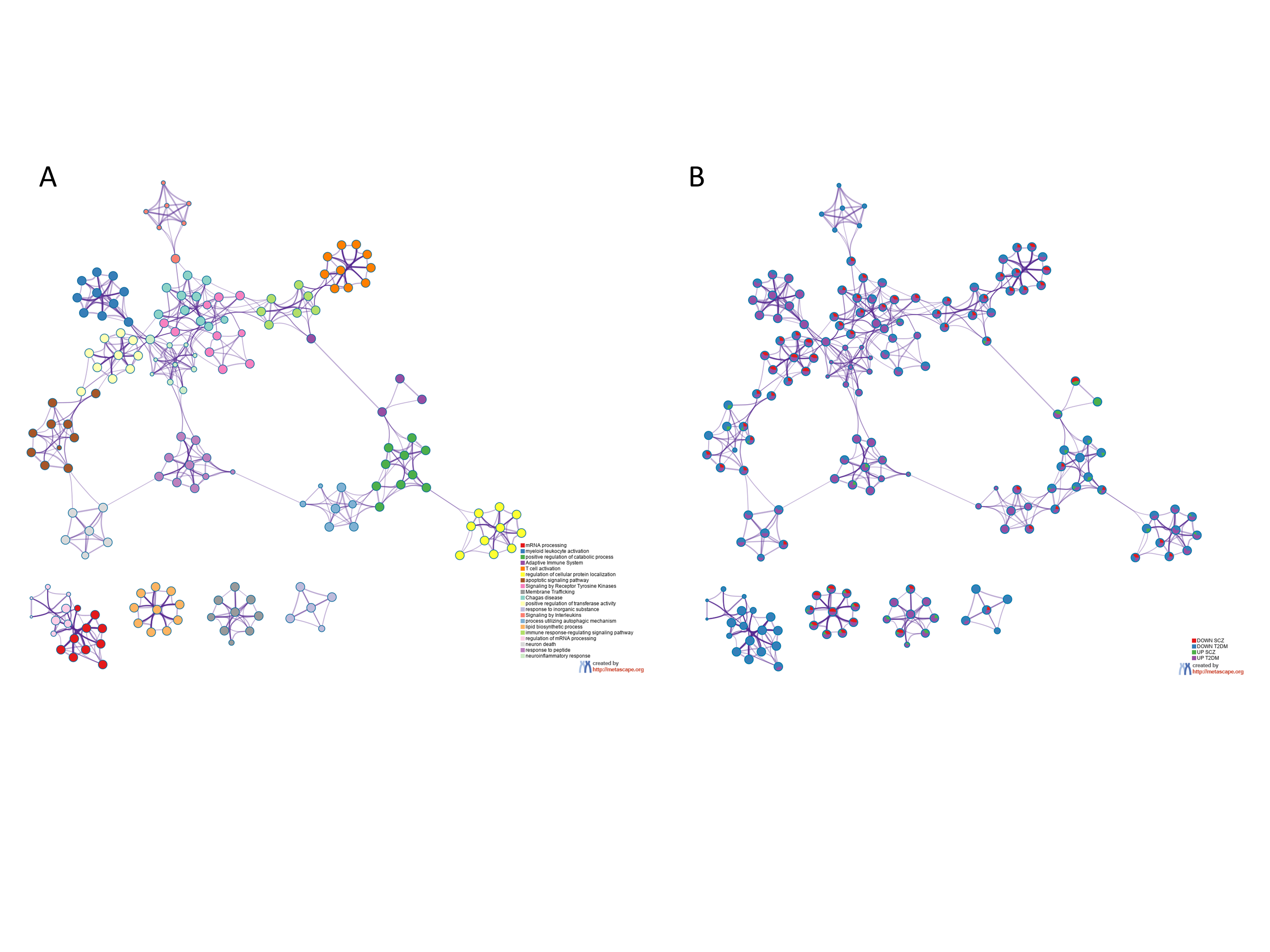

Supplement: Supplementary file 1 [file genes-12-00237-s001.zip › supplementary/Figure S1.png]
